# P143: Teaching the concepts of hand hygiene to undergraduate medical students: the views of key stakeholders

**DOI:** 10.1186/2047-2994-2-S1-P143

**Published:** 2013-06-20

**Authors:** R Kaur, H Razee, H Seale

**Affiliations:** 1 School of Public Health and Community Medicine, University of New South Wales, Sydney, Australia

## Introduction

Currently, hand hygiene (HH) compliance rates amongst Australian medical students are below 70% nationally. Previous attempts to improve medical students’ knowledge of HH have had only short-term successes with follow-up studies reporting poor long-term retention of knowledge worldwide. It has been previously suggested that the importance of HH must be taught to medical students from the first year and integrated into their clinical curricula.

## Objectives

Our study aimed to examine the current practices around teaching concepts of hand hygiene in an Australian tertiary educational institute

## Methods

In-depth interviews were conducted with a purposeful sample of key members of the undergraduate medical teaching team and a sample of medical students (year 1 to 6). Thematic analysis was undertaken on the transcripts.

## Conclusion

Teaching hand hygiene to medical students was considered challenging by our participants, as medical students do not rank the subject as ‘important’. It was suggested that medical students are resistant to be taught concepts such as communication or hand hygiene as they consider these things their personal habits. Professional modelling was considered the major barrier in increasing the HH compliance of senior medical students, as these students tend to mimic the behaviour of the senior doctors (‘role models’) regardless of all their teaching and training on HH. Assessing students on their HH knowledge and practice would motivate them to learn the concepts but would only have a short-term impact. The use of peer auditing, scenario based activities and patient feedback were considered as potential options which would reinforce the need to HH and the potential for new opportunities to teach the concepts. The Medical students interviewed rated their hand hygiene compliance as high and hence they felt they would not turn up to classes if hand hygiene were formally taught. Teaching hand hygiene as infection control within a patient safety context was considered a major motivator.

## Disclosure of interest

None declared

